# Supplementary material for: Deletion of wfs1 Impairs Oligodendrocyte Precursor Cells Dorsal Distribution and Myelination Through the wfs1-hmgcs1 Axis in Zebrafish
Source: Biology (Basel). 2026 Aug 16;15(16):1402. doi: 10.3390/biology15161402 (PMC13510151; doi:10.3390/biology15161402)
Supplement: Supplementary file 1 [file biology-15-01402-s001.zip › File S1-Transcriptome sequencing data and differentially expressed genes (DEGs) in wfs1-- zebrafish at 5 dpf/2.KEGG/wfs1KOvsWT/DOWN/src/dre04540.html]

dre04540


- dre:497384

- Down regulated genes

497384\_1(-2.05267237809407)

- dre:566430

- Down regulated genes

566430\_1(-3.52930553579608)

- dre:100000981

- Down regulated genes

100000981\_2(-1.61456337824489)

- dre:336681

- Down regulated genes

406811\_1(-1.09337424046752)

- dre:386856

- Down regulated genes

386856\_1(-1.99487147218035) novel.946(-6.7676180476835)
- dre:571431

- Down regulated genes

571431(-6.42862549702529)

- dre:560564

- Down regulated genes

560564\_2(-4.02056550705436)
- dre:103909601

- Down regulated genes

378480(-1.33145438810737)

Close
